# Natural and Synthetic Neurotoxins in Our Environment: From Alzheimer’s Disease (AD) to Autism Spectrum Disorder (ASD)

**DOI:** 10.4172/2161-0460.1000249

**Published:** 2016-07-26

**Authors:** Aileen I Pogue, Walter J Lukiw

**Affiliations:** 1 Alchem Biotech, Toronto ON M5S 1A8, Canada; 2 Neuroscience Center, Louisiana State University Health Sciences Center, New Orleans, LA 70112, USA; 3 Department of Ophthalmology, Louisiana State University Health Sciences Center, New Orleans LA 70112, USA; 4 Department of Neurology, Louisiana State University Health Sciences Center, New Orleans LA 70112, USA

**Keywords:** Aluminum, Alzheimer’s disease (AD), Aspirin, Autism spectrum disorder (ASD), Human biochemical individuality, Neurotoxic metal, Non-steroidal anti-inflammatory drugs (NSAIDs), Samter’s triad

## Overview

The earth’s population of 7.4 billion continue to live in an increasingly toxic environment filled with multiple-natural and synthetic bioactive chemicals, compounds and biologicals that can adversely affect human health and well-being [1,2]. A high proportion of these bioactive agents, often at very-low and physiologically-realistic concentrations, are toxic to the structure and function of the human central and peripheral nervous system (CNS, PNS), and hence are said to be ‘*neurotoxic’*; in addition many of these chemicals significantly disrupt the structure and function of the human genome and processes such as the read-out of genetic information from DNA, often referred to as gene expression, and hence are said to be ‘*genotoxic’*. In this ‘Short Commentary’ we will review some recent data addressing why certain human individuals or populations may be, because of their intrinsic genetic makeup, at an increased susceptibility or risk to these environmental toxins and biologicals which can induce neurological disease. Here we will address the potential contribution of aluminum to Alzheimer’s disease (AD) and/or autism spectrum disorder (ASD) wherever possible.

## The Continuing Mobilization of New Chemicals and Neurotoxins into the Biosphere

Each year in the US alone there are about 630,000 new patent applications (2015; http://www.uspto.gov/web/offices/ac/ido/oeip/taf/us_stat.htm), many of which are for novel chemical compounds useful to the chemical, pharmaceutical, agricultural, materials, mining, energy and biotechnology industrial sectors [1]. In 2015, for example, the total number of new patents, traceable as registered patent documents worldwide, was estimated to be more than 2,700,000, and of these, approximately 22% or ~ 594,000 were chemistry-, biochemistry- or medicine-related involving novel chemistries, drugs, pharmaceuticals and related chemical compounds, their biosynthesis and their technological applications [1–3]. Very often exquisitely small amounts of these novel chemical compounds are toxic at extremely low levels to CNS and PNS structure and function. Even pharmaceuticals are hepatotoxic at some level, and this is of increased concern in the elderly who have compromised hepatic function in their old age. Globally, and especially in recent years: (i) the vast majority of these new chemical compounds have not been individually tested for their neurotoxic or genotoxic properties; (ii) their detrimental effects toward human health and welfare when combinations of them are used are unknown; and (iii) their novel biological effects when they are utilized for various purposes against the vast background of already existing chemicals has not been determined. Indeed, it is a major scientific effort for environmental and/or neurotoxicological researchers to understand in detail how just one of these new compounds affects human health, let alone the often highly interactive toxic contributions from several hundred thousand. *Indeed it is well beyond the current capacity of human environmental and toxicological science to understand the combined interactive effects of how 594,000 novel chemical compounds could simultaneously interact with each other and how together they could impact human physiology, neurochemistry and neurobiology and contribute to neurological disease.* With rapidly expanding human populations and the technology-driven need for novel chemicals, including pesticides and herbicides used to enhance food production, we can expect these kinds of problems to become even more significant and complex in the foreseeable future.

## The Mobilization of Neurotoxins into the Biosphere: Focus on Aluminum

In concert with the global generation of new chemical compounds is the mobilization of normally earth-bound neurotoxins, and here we will underscore the remarkable and ongoing liberation of aluminum into the biosphere, resulting in its increased bioavailability to neurobiological systems. Not only is aluminum the most abundant naturally occurring metal in our biosphere, globally aluminum is now the world’s most widely used non-ferrous metal, and its mining and purification exceeds that of any other metal except iron [4,5]. Worldwide yearly production of primary aluminum is ~ 52 million tons or about 15 pounds for very person on the earth [4–9]. Global demand for aluminum from developing countries is increasing, due in part to new applications for aluminum and aluminum alloys for multiple applications in aviation, aerospace, munitions, electrical transmission and energy generation, in infrastructural support including construction, in transportation, packaging, and in food and medical applications [4–6,8,9]. In parallel with the massively increased bioavailability of aluminum are the increased global production and mobilization into the environment of other neurotoxic elements, gases and metals, chiefly lead, mercury, chrome, cadmium, carbon and nitric oxides and others [6,9]. This prolific generation and ‘mobilization’ of toxins into the biosphere combined with highly efficient recycling programs means these known chemical agents are becoming increasingly bioavailable. Therefore, in addition to the thousands of new chemicals being generated and released into our biosphere (the sum of all ecosystems and living organisms on the earth) we see a parallel mobilization of normally earthbound neurotoxic and genotoxic elements [8,9]. Interestingly, certain chemical compounds (such as glyoxalates) and aluminum (as, for example, in the vaccine adjuvant aluminum oxide or the common food additive aluminum maltolate) have been implicated in the development of several human neurological disorders including AD and ASD [7–16]. *Indeed aluminum’s widespread mobilization into our biosphere may place all of humankind at a heightened inflammatory status thereby increasing general risk for the development of AD, ASD and other progressive neurodegenerative disorders with an inflammatory component* [6–16].

## Human Biochemical Individuality

If we assume that ~ 23,000 human genes are available to be expressed in each cell, and that each gene is responsible for at least one genetic function, then with a global human population of 7.4 billion it becomes very difficult to assign a single ‘*standardized human gene expression profile*’ that is representative for the phenotype of the human species [17–27]. Interestingly the Coriell Institute for Medical Research selected samples from 26 different human populations to create a ‘*standardized human DNA panel*’ in the 1000 Genomes Project population sampling for the NHGRI Repository [16–18]. ‘Taken together, these ideas, in part, form the basis for (i) the still evolving concepts of ‘human genetic individuality’; (ii) our efforts to better comprehend the genotypic basis of phenotypic diversity; and (iii) how this may be important in human health and susceptibility to disease [16–19,21,24]. Large population studies have recently analyzed the potential contribution of variability in global gene expression patterns, including the impact of genetic mutations, to ‘*human genetic individuality*’, phenotype, susceptibility to disease and related genotypic parametrics [17–22]. Major recent results of these studies indicate: (i) identification of a considerable redundancy in human gene-expression on a global-scale; and (ii) the presence of environmentally- or genetically-influenced gene-expression patterns which give individuals, or certain human populations, characteristic genotypes and phenotypes [16–22]. For example recent DNA array-derived total messenger RNA and microRNA profiles from different individuals indicate that the abundance of these regulatory ribonucleic acids can significantly differ between human population groups suggesting that genetic variation and extraneous effects, including age, gender, body mass index, AD-relevant allele status, life-style and intrinsic environmental and/or epigenetic effects can strongly modulate the ultimate phenotype [20–24]. Hence, depending on genotypic or phenotypic considerations, certain individuals or population groups may be chronically predisposed to the multiple effects of neurotoxic and genotoxic agents over the course of a lifetime.

## Human Biochemical Individuality - The Example of Aspirin

One common and highly illustrative example of ‘*human biochemical individuality’* is human physiological reaction to the ingestion of aspirin (acetylsalicylic acid; ASA); the world’s most globally available analgesic, most prescribed and most common over-the-counter (OTC) globally available drug compound. Worldwide, an estimated 46,000 tons of aspirin are consumed each year [28–30]. While a small percentage of people, typically less than 1%, are directly allergic to aspirin, far greater numbers, upwards of 12%, have an allergic sensitivity to this pain killing non-steroidal anti-inflammatory drug (NSAID). In these later cases not only does aspirin have a very limited (or even non-existent) analgesic action when ingested, but rather induces many unintended effects including asthma and related pro-inflammatory respiratory complications sometimes referred to as ‘*aspirin-sensitive asthma’*, ‘*aspirin exacerbated respiratory disease*’ (AERD), *aspirin-induced asthma and rhinitis* (AIAR), collectively known as ‘*Samter’s triad*’ [28–32]. Medically, the ingestion of aspirin is in most cases beneficial to the patient through the promotion of analgesia, however about 6% of all people have some form of aspirin-sensitivity and experience *Samter’s triad* that can develop into sudden, severe respiratory distress requiring emergency medical treatment. The preferred treatment is avoidance of exposure to aspirin itself [28–32]. *The important take-home message here is that there are multiple, highly individualized responses in humans to the very same drug compound which in most instances are fairly well tolerated by the patient, but in other instances are highly refractory to the patient’s homeostatic physiology and especially their innate-immune and inflammatory status which may culminate in hospitalization. These findings may also help explain an individual’s adverse, and sometimes extremely negative reaction to, for example, the aluminum used in OTC medications or in vaccines administered with an aluminum adjuvant. Aluminum neurotoxicity can therefore be thought of in a similar fashion – that some individuals can adequately tolerate aluminum exposure, either chronic or acute, while others simply cannot.* Hence neurological diseases such as AD and ASD may be the result of the poorly understood parameters that contribute to *human biochemical individuality. Put another way the susceptibility and predisposition of an individual to ASD, AD and other environmental-synthetic neurotoxin-linked diseases may be very well based on an individual’s unique genetic, epigenetic, biochemical, and neurochemical composition and/or environmentally-based factors. These are against either (i) a background of brain development in the case of children; or (ii) over the course of aging and in the elderly when normally protective physiological barriers such as the gastrointestinal (GI) tract and blood-brain barrier (BBB) are either not fully formed or begin to break down with age, and leak environmentally-abundant neurotoxins into susceptible and sensitive biological compartments* [9–16,33–35].

## The Outlook for the Future

Each year the earth adds to its population about 81,000,000 individuals with each individual carrying the potential for the development at least 14,000 different diseases [2,3,16–18,36]. These increases in population and incidence of disease occur against a background where more and more medical research and healthcare is urgently needed yet less and less is readily available. *This alarming combination of increasing population growth that includes genetically susceptible human individuals, and increased mobilization of multiple forms of normally earthbound and synthetic neurotoxins, genotoxins, novel chemical compounds, hydrocarbons including carbon and nitrogen oxides and related greenhouse gases, and other disease-causing agents into our environment continues to overwhelm our scientific and medical capability to understand their complex environmental, genetic and epigenetic interactions and address them as potentially serious healthcare concerns*. As underscored by the recent massive outbreaks of the Ebola or Zika virus in central Africa, when and where the next ‘*environmental disease, epidemic or plague’* will appear is not known, but the certainty is that some novel health crisis will appear unpredictably, at random and/or may represent a rapid escalation of an already partially characterized disease entity. *The concept of ‘human biochemical individuality’ tells us that human individuals are genetically unique, with certain gene expression patterns or genetic compositions that may make them especially sensitive, and perhaps predisposed, to the genotoxic effects of a particular synthetic chemical compound or environmental neurotoxin. Given the appropriate incubation period in genetically sensitive hosts these exposures can ultimately contribute to AD or ASD, as well as other serious incapacitating neurological diseases of the human CNS* [6–15].

## Summary

*Humankind has inadvertently designed a remarkably precarious combination of rapidly increasing and unchecked population growth with an increased liberation of novel and potentially pathogenic chemical compounds and neurotoxins into the biosphere. Parallel increases in the deleterious consequences of unrestricted population growth and diseasecausing toxic exposures in our environment are on the horizon.* Globally this poses very significant socioeconomic and healthcare concerns that have been neglected for far too long. The perception of ‘*human biochemical individuality*’ indicates that certain individuals or human populations with specific genetic backgrounds may be predisposed to these toxic actions through either an acute or a more chronic type of life-long environmental exposure [11,33–36]. To cite one important example, since there is abundant evidence that neurotoxic compounds such as aluminum may play initiator or disease-propagating roles for AD, ASD and other progressive, age-related neurological diseases, then we should expect these kinds of exposure situations that can adversely affect human health and welfare to become even more common and widespread in the foreseeable future [7–27]. This may be particularly important socioeconomically and epidemiologically due in part to the excessive and additional burden it will place on our already strained healthcare system both here in the United States and in global situations where even basic healthcare systems remain underdeveloped or are simply unavailable to the local human population.
